# Quantitative Evaluation of Debris Removal from NiTi Rotary Endodontic Instruments After Different Cleaning Procedures

**DOI:** 10.3390/dj13020049

**Published:** 2025-01-23

**Authors:** Luigi Generali, Paolo Generali, Pio Bertani, Francesco Cavani, Vittorio Checchi, Tommaso Filippini, Federica Veneri

**Affiliations:** 1 Department of Surgery, Medicine, Dentistry and Morphological Sciences with Transplant Surgery, Oncology and Regenerative Medicine Relevance (CHIMOMO), University of Modena and Reggio Emilia, 41124 Modena, Italy; luigi.generali@unimore.it (L.G.); paolo.generali@unimore.it (P.G.); vittorio.checchi@unimore.it (V.C.); federica.veneri@unimore.it (F.V.); 2 Private Practice, 43121 Parma, Italy; segreteria@piobertani.com; 3 Department of Biomedical, Metabolic and Neural Sciences, University of Modena and Reggio Emilia, 41124 Modena, Italy; francesco.cavani@unimore.it; 4 School of Public Health, University of California Berkeley, Berkeley, CA 94704, USA

**Keywords:** debris, decontamination, cleaning, endodontic files, NiTi, SEM

## Abstract

**Objectives:** Endodontic instruments require thorough decontamination and sterilization before use and reuse to ensure the safety and success of treatments. However, standardized protocols are lacking. This study aimed to quantitatively assess the effectiveness of different cleaning protocols in removing debris from NiTi rotary file surfaces. **Methods:** Forty-eight new Mtwo NiTi rotary instruments (sizes 10/.04, 15/.05, 20/.06, and 25/.06) were randomly assigned to four groups (n = 12). A set of new sterile instruments (Group I) served as the negative control. After usage for primary endodontic treatment, instruments underwent different cleaning protocols: steam sterilization without cleaning (Group II); ultrasonic cleaning + steam sterilization (Group III); and manual cleaning with a scouring sponge + ultrasonic cleaning + steam sterilization (Group IV). Back-scattered scanning electron microscopy (SEM) micrographs of the apical, middle, and coronal sections were processed using Fiji software (version 2.14.0) to quantify debris as a percentage of the total selected area. **Results:** No significant differences were found among the three sections within each group, although higher debris amounts were observed from coronal to apical in Groups I and II. Group I had the least debris, while Group II showed the most, with statistically significant differences compared to other groups (*p* < 0.05). There was no significant difference between Groups III and IV, though Group IV showed notably less debris. **Conclusions:** The combination of mechanical, chemical, and ultrasonic cleaning proved most effective at removing debris from endodontic instruments. Current cleaning methods, however, remain insufficient for complete debris removal, highlighting the need for further research to standardize and improve cleaning and sterilization protocols or preferably use single-use/single-patient instruments.

## 1. Introduction

Nickel–titanium (NiTi) rotary files have significantly improved root canal preparation, reducing the risk of complications compared to hand instruments [1,2,3]. Numerous studies have demonstrated that NiTi instruments can be reused multiple times without significantly increasing the risk of fracture [4,5,6]. On the other hand, manufacturers recommend discarding these instruments after a specific number of use cycles or whenever deformations can be observed in order to prevent unexpected fractures during root canal therapy [7,8]. Some NiTi instruments are, however, recommended for a single use only due to concerns about adequately removing organic debris after cleaning procedures [9,10].

Endodontic treatment can result in contact between instruments and blood, pulp tissue, and potential microbial contaminants, with organic and inorganic materials accumulating along the instruments’ flutes and surfaces. Contaminated instruments can transmit bacteria and viruses between patients, as well as prion-based diseases such as Creutzfeldt–Jakob disease [11,12,13].

Moreover, decontamination and sterilization procedures may expose healthcare personnel to a risk of infection, particularly when handling sharp instruments. For these reasons, endodontic instruments are classified as “critical items” that require thorough cleaning and sterilization before use [14].

Procedures for controlling cross-infections in dental settings have significantly changed daily clinical practice. The reuse of endodontic instruments involves several cleaning procedures to remove organic debris, followed by sterilization [15,16]. Organic debris can compromise the effectiveness of disinfectants by preventing their contact with the instrument surface or by binding to the molecules of chemical disinfectants, making them inactive [17,18]. Moreover, biological debris may prevent the proper penetration of the sterilization steam [9,19]. However, cleaning endodontic instruments is extremely challenging due to their complex surface topography and small size [12].

Several approaches have been implemented for the debridement of endodontic files, including mechanical cleaning using brushes and sponges and chemical cleaning through immersion in solutions such as sodium hypochlorite at different concentrations, hydrogen peroxide, detergents, and enzymatic solutions. Other methods involve combining mechanical and chemical cleaning or utilizing ultrasonic systems for enhanced debris removal [15].

However, certain chemical agents can be detrimental to metal surfaces, potentially leading to significant damage, and the corrosion of NiTi files has been reported [20,21]. Furthermore, they are unable to eliminate organic debris from used endodontic instruments completely [22].

Ultrasonic cleaning is one of the most widely used methods for debriding endodontic instruments. Instruments are immersed in a cleaning solution, in which ultrasonic waves create cavitation bubbles that displace organic and inorganic debris from the surface. This method is effective for accessing the complex geometries of the instruments, ensuring thorough cleaning [12].

However, both manual debridement of instruments and ultrasonic cleaning alone are not able to completely remove debris from rotary NiTi [23,24]. Ultrasonication has thus been combined with vigorous manual strokes using a sponge, along with pre-soaking in an enzymatic solution in order to improve instrument cleaning [25,26]. This combined protocol yielded encouraging results in both laboratory and clinical settings in removing most organic debris from instruments [24,25].

Briefly, Linsuwanont et al. proposed the following cleaning sequence: brushing the instruments with a nylon brush for 20 strokes; immersion in 1% sodium hypochlorite (NaOCl) for 10 min; and finally, ultrasonic bath for 5 min [24]. NaOCl 0.5% to 5.5% is considered an effective disinfectant for endodontic use due to its antimicrobial activity and its ability to dissolve organic material [27,28]. Furthermore, it is not associated with significant corrosive effects on NiTi instruments, even after prolonged contact for up to 48 h [29,30]. However, since the use of NaOCl directly in the ultrasonic bath is not feasible due to its corrosive effect on the device, and utilizing glass containers filled with NaOCl placed in the ultrasonic bath significantly reduces the disinfectant’s efficacy, the use of enzymatic cleaning solutions has been recommended, as this method appears to have equivalent efficacy without risking equipment deterioration [10,25].

Preliminary mechanical cleaning procedures are also crucial, and the use of nylon brushes, gauzes, or sponges for scrubbing the instrument surface has been proposed as a common cleaning step. However, this manual cleaning phase is associated with a higher occupational risk of infection [25].

Many authors have debated the efficacy of different methods and protocols for cleaning and removing debris from the surface of endodontic instruments after use [15,26,31].

Research on effective cleaning protocols has seen minimal progress over the past two decades. This is evidenced by the lack of standardized procedures and limited innovation in this field. For example, studies have reported great variability in sterilization and cleaning practices for endodontic instruments, which has been linked to inconsistent effectiveness and adverse effects on instrument properties after repeated sterilization cycles [32,33]. As a result, there is currently no standardized protocols and consistent information on the optimal cleaning procedures for reusable endodontic instruments [9,23,34].

Therefore, this study aimed to quantitatively evaluate the presence of surface debris on reusable NiTi rotary endodontic instruments, processed by different cleaning protocols before steam sterilization, by scanning electron microscopy (SEM) imaging. The null hypothesis tested was that the different cleaning procedures investigated would not significantly influence the amount of debris remaining on the instrument surface.

## 2. Materials and Methods

### 2.1. Experimental Layout

Forty-eight new Mtwo (Sweden & Martina, Due Carrare, Padova, Italy) NiTi rotary instruments sized 10/.04, 15/.05, 20/.06, and 25/.06 were used, with a working part of 16 mm. Each instrument was inspected under an optical microscope (OPMI Pico; Carl Zeiss Meditec Inc., Jena, Germany) at 10× for defects before the experiment. Since no defects were detected, no instrument was discarded. Instruments were randomized by a computer algorithm (www.random.org, accessed on 18 January 2024) into 4 groups of n = 12 instruments each (three sets of four files No. #10–25), which underwent different cleaning procedures.

The study groups were defined as follows: Group I—new sterile instruments (negative control); Group II—steam sterilization without cleaning; Group III—ultrasonic cleaning and steam sterilization; and Group IV—scouring sponge, ultrasonic cleaning and steam sterilization.

### 2.2. Endodontic Treatment

Each set of instruments was used to shape four root canals of the upper molars in nine patients presenting at the urgent dental care service of the Dental Clinic of the Policlinico di Modena University Hospital in Modena (Italy), with an indication for primary endodontic therapy. The clinical diagnosis for all nine teeth was deep caries associated with pulp necrosis, which was made following a negative response to the cold vitality test performed with a hydrogen fluorocarbonate spray. The access cavity was then opened without local anesthesia, confirming the absence of vitality. No radiographic evidence of periapical lesions was observed in any of the patients. No signs of pus drainage were observed.

After gaining access to the pulp chamber, the canal orifices were located, and the root canals were negotiated using size 08 and 10 stainless steel files (K-file, Dentsply Maillefer, Ballaigues, Switzerland) lubricated with ethylenediaminetetraacetic acid (EDTA) at 15% and 10% urea peroxide cream. The working length (WL) was determined using an electronic apex locator (Apex ID, SybronEndo Kerr Dental, Brea, CA, USA). The recorded WLs were in a 21 ± 1 mm range. Shaping was performed with Mtwo (VDW, Munich, Germany) NiTi rotary instruments in the following sequence: 10/.04, 15/.05, 20/.06, and 25/.06, operated at 280 rpm and 1.2 to 2.3 Ncm, according to the manufacturer’s recommendation, using an X-Smart Plus (Dentsply Sirona, Ballaigues, Switzerland) endodontic motor. Irrigation was performed alternating 17% EDTA and 5.25% NaOCl between each instrument.

After each passage in the canal, the instrument was inserted into a sponge soaked in ethyl alcohol to remove coarse debris that remained on the instrument’s surface. The apical diameter was determined, the WL was confirmed with the electronic apex locator, and canal obturation was performed using Thermafil (Dentsply Sirona) obturators and Top Seal (Dentsply Maillefer) sealer after drying the root canals with sterile absorbent paper points.

### 2.3. Cleaning Procedures

In Group I (new sterile instruments), instruments were carefully removed from the manufacturer’s packaging using tweezers to avoid touching and contaminating the tips and cutting flutes. Each instrument was mounted on a metallic stub and observed with a scanning electron microscope (ESEM—Quanta 200, Fei Company—Oxford Instruments, Eindhoven, The Netherlands) to evaluate the presence on the surface of any manufacturing residues. These instruments were not used for shaping procedures; thus, they did not undergo any cleaning procedure and served as the negative controls.

In Group II (steam sterilization without cleaning), the instruments were wrapped in porous paper and autoclaved at 134 °C for 20 min (immediately after the shaping procedure). The instruments of this group served as the positive control.

In Group III (ultrasonic cleaning and steam sterilization), the instruments were placed in a wire mesh basket within 30 min of use and immersed in a disinfectant solution for 30 min (Sekusept^®^ Pulver classic, Ecolab, Vimercate, MB, Italy). The wire mesh basket containing the instruments was then placed for 15 min in an ultrasonic bath containing an enzymatic cleansing solution (Septozym CE, Nuova Farmec s.r.l., Settimo di Pescantina, Verona, Italy). Instruments were then drained and rinsed in demineralized water for 20 s and finally autoclaved at 134 °C for 20 min.

In Group IV (scouring sponge, ultrasonic cleaning, and steam sterilization), the instruments were placed in a wire mesh basket within 30 min of use and immersed in a disinfectant solution for 30 min (Sekusept^®^ Pulver classic, Ecolab), as in Group III. Subsequently, instruments were manually cleaned by inserting them in a scouring sponge with an in-and-out movement for 10 consecutive times. As in Group III, the wire mesh basket containing the instruments was then placed for 15 min in an ultrasonic bath containing an enzymatic cleansing solution (Septozym CE, Nuova Farmec s.r.l.). Instruments were then drained and rinsed in demineralized water for 20 s and finally autoclaved at 134 °C for 20 min.

### 2.4. SEM Imaging and Debris Quantification

During all procedures, instruments were handled with tweezers to avoid contact with the tip and the cutting flutes. The instruments were mounted horizontally on metallic stubs with adhesive conductive tape and examined under a scanning electron microscope (ESEM—Quanta 200, Fei Company—Oxford Instruments, Eindhoven, The Netherlands). In order to standardize the observations, all instruments were mounted with the main slot of the shank parallel to the stub. SEM imaging was performed on the single exposed surface of each instrument to avoid potential bias in the observations of possible overlapping surfaces due to the round shape of the instruments, and to avoid multiple manipulation and subsequent possible contamination. This approach ensures the reproducibility of the analysis and was also adopted in line with another similar study [9]. Micrographs were taken in backscattered mode to discriminate the metallic nature of the file from the debris. In particular, the debris appears dark compared to the brighter appearance of the metal surface.

Three 2 mm sections (apical, middle, and coronal) of each instrument were examined, and for each section, two sequential 1 mm length micrographs were obtained at 270× magnification [9]. A total of 96 micrographs were obtained, saved as digital images, and processed using Fiji software (National Institutes of Health, Bethesda, MD, USA).

**Figure 1:**
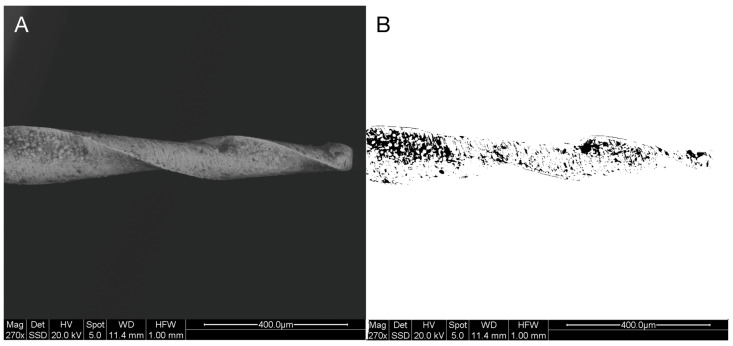
Debris quantification through the processing of SEM images (**A**) using Fiji software (National Institutes of Health, Bethesda, MD, USA). The “threshold” function was used to obtain a binary image and highlight the dark spots (**B**), which were then measured using the “measure” function. The debris amount was expressed as the percentage of dark spots over the total selected area (ROI), defined as the contour of the file.

Briefly, the contour of the file was outlined, thus defining the Region Of Interest (ROI). Within this ROI, the dark spots were selected using the “threshold” function to obtain a binary image, which was then measured using the “measure” function. In cases where dark shades were present due to the file’s morphology, these were removed using the “subtract background” function (Figure 1). The debris amount was expressed as the percentage of dark spots over the total selected area (ROI).

### 2.5. Statistical Analysis

Percentages of the debris were summarized by calculating, separately for each study group, the mean and standard deviation (SD) for each file section (apical, middle, and coronal) and the entire files. After assessing the normality of the distribution using the Shapiro–Wilk test, the non-parametric Kruskal–Wallis test, followed by Dunn’s post hoc test when appropriate, was used to detect significant differences among groups and root regions. A *p* < 0.05 was considered statistically significant. Statistical analyses were performed using Stata 16.1 software (StataCorp LCC, College Station, TX, USA).

## 3. Results

### Debris Quantification

**Figure 2:**
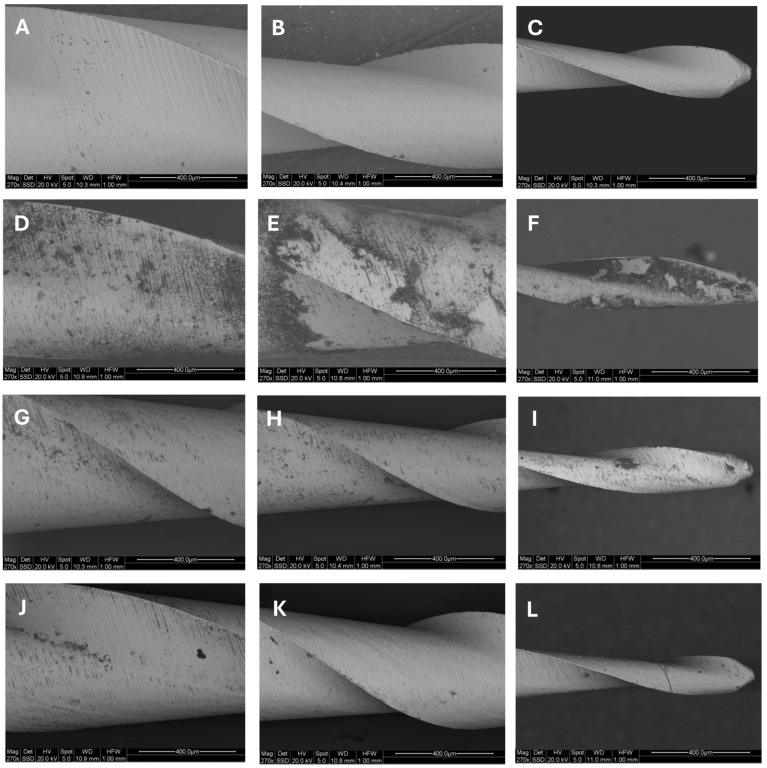
Representative images of SEM back-scattered acquisitions at 270× magnification of instruments’ sections from Group I (new instruments; (**A**) coronal, (**B**) middle, and (**C**) apical), Group II (uncleaned, autoclaved instruments; (**D**) coronal, (**E**) middle, and (**F**) apical), Group III (ultrasonic cleaning and steam sterilization; (**G**) coronal, (**H**) middle, and (**I**) apical), and Group IV (scouring sponge, ultrasonic cleaning, and steam sterilization; (**J**) coronal, (**K**) middle, and (**L**) apical).

Table 1 reports percentages, expressed as mean values ± SD, of the amount of debris measured in each third and for the entire file in each group. Within each group, no significant differences were found among the three examined regions, despite a trend that could be observed in Groups I and II, where the apical thirds showed a greater amount of debris compared to the coronal ones. The comparison among groups revealed that new files (Group I) had a significantly lower amount of debris compared to all the other groups, both when the entire file or each third was considered. On the other hand, uncleaned instruments (Group II) showed the highest amount of debris compared to the other groups, and the differences were always statistically significant, regardless of whether the entire file or each third was considered. Ultrasound-cleaned (Group III) and sponge-soaked instruments (Group IV) showed similar percentages of debris, without significant differences between each other (*p* < 0.05). Representative images of SEM acquisitions are displayed in Figure 2.

## 4. Discussion

The unnecessary disposal of rotary NiTi files after a single use can significantly influence the overall cost of a root canal treatment for both dentists and patients [35]. Most NiTi rotary files, including those investigated in the present study, are designed to be reused multiple times. Therefore, an effective cleaning and sterilizing treatment is strictly required, as indicated by manufacturers providing specific reprocessing protocols [36].

There is currently no consensus on the optimal disinfection protocols and the efficacy of different cleaning methods [12].

In dental practices, various methods are employed for cleaning instruments prior to sterilization, yet universally accepted standard cleaning procedures have not been established [15]. The present study compared the amount of debris observed in new unused instruments and instruments undergoing different cleaning protocols.

The methods analyzed in this study varied significantly in key aspects, including the type of disinfectant used, the soaking time, the duration of the ultrasonic bath and the solvent used, and whether mechanical cleaning was incorporated into the process.

In the present study, a prolonged pre-soaking time of 30 min in a peracetic acid-based disinfectant solution and a shorter ultrasonication time of 15 min were employed, following the recommendations from previous research [24,25,36]. Notably, no signs of corrosion were observed on the NiTi instruments, confirming the absence of detrimental effects from the selected chemical agents.

The null hypothesis was rejected, as significant differences were found in the amount of debris remaining on the instruments’ surfaces following the different cleaning procedures tested.

New instruments showed less than 1% debris on their surface. This observation aligns with previous studies, confirming that new unused files, even when taken directly from their package, are not sterile. Trace amounts of both inorganic and organic debris have been detected on their surfaces [12,18,22,24,34]. These contaminants can originate from multiple sources, including manufacturing, packaging, and even cleaning processes [19]. Reports have identified adherent deposits containing carbon, sulfur, and silica-based particles, which are believed to result from lubricating oils and refining steps during production [24,37]. Although the clinical significance of such residues remains unclear, they could act as irritants, antigens, or infective agents. Moreover, these deposits may facilitate additional debris accumulation during clinical use, potentially impacting the instrument’s performance and safety [18].

Used uncleaned (only autoclaved) files have 33% of their surface covered with debris. This value is significantly higher compared to unused sterile files, as anticipated. This confirms that a large amount of debris, in particular the presence of biological debris, prevents the proper penetration and disrupting action of the sterilization steam, making autoclave treatment alone insufficiently effective [9,19].

Ultrasonic cleaning resulted in the removal of most of the debris. On average, the amount of debris found in ultrasonicated files is 5.6%, indicating a significant reduction compared to uncleaned files.

When ultrasonic cleaning is additionally preceded by soaking in a scouring sponge, the average amount of surface debris further decreases to 2.6%. While this difference is not statistically significant, it is noteworthy that this value is half of that observed in instruments only cleaned by ultrasonic bath. Despite the lack of significance that could be attributed to the small sample size in this study, resulting in wide standard deviations, this finding may have a clinical relevance. Similar results and considerations can be applied to different sections when each instrument’s third is considered. None of the decontamination protocols used in this study were, however, entirely effective in removing debris, as also reported by most similar studies [18,34,38]. When combined with ultrasonication, these detergents deliver notable results [25]. The synergistic effect of cavitation from the ultrasonic bath and the chemical activity of the enzymatic detergent significantly enhances debridement [25]. Overall, these findings suggest that the mechanical action of manual brushing plays a crucial role in improving debris removal, thus confirming its importance as a pivotal step in disinfection and sterilization protocols of endodontic instruments [15,31].

Interestingly, an increasing trend of surface debris, although not statistically significant, was observed in the coronal–apical direction of new and uncleaned instruments. This suggests that the apical third of the files tend to be more contaminated after endodontic treatment, regardless of the working length, which was similar for all the used instruments and also always involved the coronal sections of the instruments. Also, although the clinical diagnosis for all patients was of pulp necrosis, the greater amount of debris found in the apical third may indicate the presence of pulp residuals or infected demineralized dentin in this area. In contrast, this trend was not observed in instruments that underwent a cleaning procedure as they exhibited a similar amount of debris across all three sections. This indicates that even in the most contaminated regions, an effective debridement is achieved through the cleaning method tested.

A notable strength of this study lies in its quantitative evaluation of debris removal.

Conversely, many other studies mostly conducted semi-quantitative or semi-qualitative assessments based on visual scores, which often lack a clear definition [23,31,38,39,40]. While many studies employed staining techniques, such as Van Gieson’s stain, to differentiate between organic and inorganic debris [12,18,24,25,34,41,42], few utilized quantitative assessments like SEM analysis for surface debris evaluation. One exception is Eldik et al., whose methodology this study partially adopted, who also examined a greater length of each file compared to most previous studies, offering a more comprehensive analysis of biological debris distribution across the instrument sections [9]. In fact, a distinctive feature of this research is that debris distribution across different instrument regions (coronal, middle, and apical thirds) was assessed, allowing for a more accurate interpretation of contamination patterns. Our findings showed that the apical third was the most contaminated region after clinical use, likely due to the specific dynamics of the clinical procedure. This underscores the need for enhanced cleaning protocols targeting this critical area to ensure thorough debridement.

While providing valuable insights, this study does have some limitations. One key limitation is the limited number of variables tested in the cleaning protocols. Specifically, this study did not explore the full range of disinfectant types, concentrations, processing times, or the characteristics of manual scrubbing, nor did it compare different combinations of these factors, as seen in other studies [23]. Such comparisons could provide a more comprehensive understanding of how these variables interact and influence the cleaning efficacy of endodontic instruments.

Another limitation is that, while this study focused on the removal of physical debris, it did not assess the potential presence of viable microorganisms or their pathogenicity, which could significantly impact the clinical relevance of the findings. Such microbiological evaluations could help better understand the potential for transmitting infections through contaminated instruments. Including microbiological analyses in future research could provide a more holistic view of the effectiveness of cleaning protocols in preventing cross-contamination and ensuring patient safety.

Further research in this field is essential, as improving decontamination methods in routine dental practice is critical for ensuring optimal instrument hygiene. Establishing a standard protocol that integrates mechanical, chemical, and ultrasonic cleaning techniques can enable the thorough removal of biological debris from endodontic instruments.

## 5. Conclusions

Ensuring complete debris removal, whether for new instruments or those intended for reuse, is essential to enhance the safety and performance of NiTi instruments in clinical practice. Nevertheless, current cleaning methods remain insufficient for removing debris completely, and trace amounts of debris were found even on new unused endodontic instruments. These findings emphasize the need for further research aimed at developing and standardizing more effective cleaning and sterilization protocols. The reduction in and the absence of debris on reusable instruments might have a positive impact on the success rate of endodontic treatments.

Within the limitations of this study, our results suggest that a combined protocol integrating mechanical, chemical, and ultrasonic cleaning procedures provides the most effective debridement of endodontic instruments, supporting the recommendation for adopting such comprehensive methods. Alternatively, it is preferable to use single-use/single-patient instruments.

## Figures and Tables

**Table 1:** Percentages of the amount of debris measured for each third and on the entire file surface in each group of files (mean ± SD).

Percentage of Debris (Mean % ± SD)				
	Group I	Group II	Group III	Group IV
Coronal	0.3 ± 0.2 ^a^	19.9 ± 21.6 ^b^	6.1 ± 4.4 ^c^	2.3 ± 2 ^c^
Middle	0.4 ± 0.2 ^a^	33.2 ± 27.7 ^b^	3.8 ± 3.3 ^c^	2.7 ± 1.7 ^c^
Apical	0.7 ± 0.5 ^a^	45.1 ± 15.3 ^b^	6.8 ± 5.1 ^c^	2.8 ± 1.3 ^c^
Total	0.5 ± 0.4 ^a^	33.3 ± 23.5 ^b^	5.6 ± 4.4 ^c^	2.6 ± 1.6 ^c^

SD: standard deviation. Different superscript letters indicate significant differences between groups (*p* < 0.05, Kruskal–Wallis test, followed by Dunn’s post hoc test).

## Data Availability

The original contributions presented in this study are included in the article. Further inquiries can be directed to the corresponding author.
